# Hemodynamic profiling by critical care echocardiography could be more accurate than invasive techniques and help identify targets for treatment

**DOI:** 10.1038/s41598-022-11252-2

**Published:** 2022-05-03

**Authors:** Stefan Schmidt, Jana-Katharina Dieks, Michael Quintel, Onnen Moerer

**Affiliations:** 1 Department of Anesthesiology, Emergency and Intensive Care Medicine, University Medical Center, Georg-August-University Göttingen, Robert-Koch-Str. 40, 37075 Goettingen, Germany; 2 Department of Pediatric Cardiology and Intensive Care Medicine, University Medical Center, Georg-August-University Göttingen, Robert-Koch-Str. 40, 37075 Goettingen, Germany

**Keywords:** Heart failure, Echocardiography

## Abstract

In this prospective observational study, non-invasive critical care echocardiography (CCE) was used to obtain quantitative hemodynamic parameters in 107 intensive care unit (ICU) patients; the parameters were then visualized in a novel web graph approach to increase the understanding and impact of CCE abnormalities, as an alternative to thermodilution techniques. Visualizing the CCE hemodynamic data in six-dimensional web graph plots was feasible in almost all ICU patients. In 23.1% of patients, significant tricuspid regurgitation prevented correlation between thermodilution techniques and echocardiographic hemodynamics. Two parameters of longitudinal right ventricular function (TAPSE and S’) did not correlate in ICU patients. Clinical surrogate parameters of hemodynamic compromise did not correlate with measured hemodynamics. 26.2% of the patients with mean arterial pressures above 60 mmHg had cardiac indices (CI) below 2.5 L min^−1^·m^−2^. A CI below 2.2 L·min^−1^·m^−2^ was associated with a significant ICU survival disadvantage. CCE was feasible in addition or as an alternative to thermodilution techniques for the hemodynamic evaluation of ICU patients. Six-dimensional web graph plots visualized the hemodynamic states and were especially useful in conditions in which thermodilution methods were not reliable. Hemodynamic CCE identified patients with previously unknown low CI, which correlated with a higher ICU mortality.

## Introduction

The invention of the pulmonary artery catheter (PAC) by Swan and Ganz in 1970 made it possible to measure extended individual hemodynamics in the intensive care unit (ICU)^1^. Following its introduction, the PAC was increasingly employed in the hemodynamic assessment of critically ill patients until studies revealed that patients in whom a PAC was used were at risk of serious adverse events and even adverse outcomes, such as an increased 30-day mortality rate^2^. There is a multitude of reasons for these adverse effects, which include interpretation problems^3^, data acquisition problems^4^, and the difficulty in translating the data into treatment decisions^5^. Despite a number of disadvantages, the PAC is still in use today^6^. However, in many European countries, transpulmonary thermodilution techniques and pulse contour analysis, such as that used by the PiCCO system, have replaced the PAC for a variety of purposes^7^. All currently available techniques and systems for assessing hemodynamics have a number of relevant limitations and contraindications. PAC measurements are, for example, inaccurate in patients with significant tricuspid or pulmonary valve regurgitation^8,9^. Pulse contour analysis is inaccurate in patients with significant tricuspid valve regurgitation, arrhythmias or pulmonary edema^10^. To overcome these disadvantages, especially in critically ill patients, there is need for alternative methods that would ideally be non-invasive, measure continuously and be feasible in situations where current thermodilution techniques have limitations and contraindications.

Critical care echocardiography (CCE) meets two of these requirements. However, this technique has previously been used only descriptively to identify cardiac abnormalities such as the presence or degree of aortic stenosis. Its ability and potential for obtaining hemodynamic measurements in the critical care environment has neither been routinely assessed nor quantified precisely.

As early as 1983, Huntsman et al. had demonstrated a close correlation between cardiac output (CO) determined by echocardiography and PAC measurements^11^. Since then, many more echocardiographic techniques for assessing hemodynamic values have been evaluated in non-critical care settings. It has been shown that in cardiac intensive care patients, non-invasive two-dimensional and Doppler echocardiographic parameters improve risk stratification for hospital mortality^12^. In detail, Jentzer and colleagues presented work suggesting that echocardiographic hemodynamic assessment in cardiac ICU patients allows not only a more accurate categorization of the clinical shock phenotype but also potentially allows a more individualized therapeutic approach^13^. In another recent study, in the majority of non-cardiologic surgical ICU patients, significant or critical cardiac abnormalities were detected that are associated with a higher ICU mortality^14^.

In this study, our first aim was to combine validated techniques of non-invasive echocardiographic measurement of hemodynamic values and evaluate the feasibility of a hemodynamic CCE protocol without the need for invasive hemodynamic techniques.

The second aim of our study was to develop and evaluate hemodynamic profiles visualized as six-dimensional web graph plots that easily allow identification of individual therapeutic targets in order to specifically improve deteriorating hemodynamic states in a particular patient.

Furthermore, the third aim of our study was to assess the impact of descriptive structural echocardiographic parameters on quantitative hemodynamic states.

## Methods

### Institutional approval, patient population and study setting

This prospective observational study was conducted in 107 consecutive patients admitted to two anesthesiology-supervised ICUs in a tertiary care university hospital. Written informed consent was obtained from all patients. Only patients with a non-cardiological, non-cardiothoracic admission diagnosis, who had not undergone recent cardiac surgery, were eligible for the study. The patient population mainly consisted of postoperative patients of any discipline, trauma patients, and patients with sepsis or acute respiratory distress syndrome. The echocardiographic assessment was conducted on the third day of the ICU stay in order to exclude postoperative patients who were admitted simply for observational reasons. The study protocol was reviewed and approved by the local ethics committee (ethics proposal Universitaetsmedizin Goettingen [UMG 11/12/13], 18/02/2014). All methods were performed in accordance with the relevant guidelines and regulations and in accordance with the amended Declaration of Helsinki; the protocol and the primary and secondary aims of the study were registered in the German Clinical Trials Register (DRKS00010208, 25/04/2016).

### Echocardiographic technique, calculation of hemodynamic parameters and hemodynamic profiling

Transthoracic echocardiography was conducted according to the recommendations and guidelines of the American Society of Echocardiography^15–19^. If these were not applicable, guidelines of the European Association of Cardiovascular Imaging were used^20,21^. In the event that neither guideline was applicable, or if threshold values were not defined, the echocardiographic techniques or values are explicitly described in the text below.

Hemodynamic evaluation was performed according to established^19^ and separately evaluated techniques as follows: Mean arterial pressure (MAP) was measured invasively via an indwelling arterial catheter. If none was in place, blood pressure was measured with an oscillometric device. Central venous pressure (CVP) was measured with a central venous catheter, or if not available, approximated from the respiratory-induced changes in inferior vena cava diameter^16^. Stroke volume (SV) and CO were determined as described by Huntsman et al.^11^ (SV = π·(LVOT / 2)^2^·VTI_LVOT_; CO = (SV·HR) / 1000; LVOT: left ventricular outflow tract; VTI: velocity–time integral; HR: heart rate). Cardiac index (CI) was calculated by the CO divided by the body surface area (BSA); BSA was determined by the Du Bois formula (BSA = 0.007184·W^0.425^·Ht^0.725^; W: weight; Ht: height). Systemic vascular resistance (SVR) was calculated using the formula SVR = (80·[MAP – CVP]) / CO. In the calculation of the systemic vascular resistance index (SVRI), CI replaced CO in the formula. Left atrial pressure (LAP) was estimated by evaluating diastolic function parameters according to previously published guidelines^18^. Mean pulmonary capillary wedge pressure (PCWP) was estimated using the Nagueh formula^22^ (PCWP = 1.24·[E / average e´] + 1.9). Pulmonary vascular resistance (PVR) was calculated according to the Abbas equation (PVR = TRV / VTI_RVOT_·10 + 0.16; TRV: tricuspid regurgitation velocity; VTI_RVOT_: velocity–time integral of the right ventricular outflow tract)^23^. Wood Units (WU) were converted to dyn·s·cm^-5^ by multiplying by 80. Systolic pulmonary artery pressure (sPAP) was approximated according to the formula sPAP = (4·TRV^2^) + RAP (RAP: right atrial pressure)^24^. In the majority of cases, RAP was measured through an indwelling central venous catheter or estimated based on the size of the inferior vena cava and its collapsibility^16,25^.

Stroke volume, CI, PVR, sPAP, SVRI and PCWP were plotted in a six-dimensional web chart to enable a rapid understanding of the hemodynamic situation. Various hemodynamic states can be easily recognized by using this visualization technique. We refer to this combined visualization and recognition technique as *hemodynamic profiling*.

The same expert CCE examiner performed all examinations. This examiner is trained in cardiology, anesthesiology and intensive care medicine, and was therefore particularly qualified to directly integrate the findings into changes in therapeutic management.

A General Electric (GE) Healthcare Vivid S5 machine equipped with a phased array adult 1.5–3.6 MHz sector scanner was used for the study. All necessary Doppler features (CD, PW, CW and TDI), imaging modalities (2D and M-mode) and software features (proximal isovelocity surface area (PISA) etc.) were available. Images were stored digitally and analyzed immediately after each examination.

### Data handling and statistical analysis

The data were pseudonymized, digitalized and further processed using Microsoft Excel (version 2013, USA). Descriptive statistics were computed in Microsoft Excel. Sigmaplot (version 12.5, Systat Software Inc., USA) and OriginPro (version 9.2, OriginLab Corporation, USA) were employed for more complex statistical analysis. Quantitative values were compared by independent *t*-tests. Kaplan–Meier curves were used for survival analysis, and groups were compared by the log-rank test. Gaussian distribution was assessed by the Shapiro–Wilk test; associations and correlations were evaluated by linear regression analysis and reported with the Pearson correlation coefficient (Pearson's *r*), the coefficient of determination (*r*^*2*^) and adjusted r-squared values (*adj. r*^2^). All regression equations are reported within the relevant figures. Spearman’s correlations and 95% confidence intervals were performed between all indices. Two-tailed *p-*values are reported; a *p*-value below 0.05 was considered statistically significant.

## Results

### Patient characteristics

Patient characteristics and details on catecholamine therapy are given in Table 1.

**Table 1:** Patient characteristics.

Mean age (years)	67.4 ± 12.6
**Sex**	
Male	73 (68.2)
Female	34 (31.8)
Mean body mass index (kg·m^−2^)	27.1 ± 5.2
Body mass index > 30 kg·m^-2^	9 (17.8)
**Outcome**	
Survived	84 (78.5)
Died	23 (21.5)
Mean length of stay in ICU (days)	12.4 ± 8.2
Required mechanical ventilation	73 (68.2)
Mean SAPS II score	33.3 ± 10.9
**Position for echocardiogram**	
Left lateral	26 (24.3)
Supine	81 (75.7)
**Left ventricular ejection fraction**	
Reduced (30–54.9%)	30 (28)
Severely reduced (< 30%)	5 (4.7)
Required catecholamine therapy	42 (39.25)
**Catecholamine received (multiple drugs possible)**	
Norepinephrine	37 (88.1)
Dobutamine	13 (40)
Epinephrine	3 (7.1)

Numerical variables are expressed as mean (± standard deviation), categorical variables as total number (percentage).

### Non-invasive hemodynamics

**Figure 1:**
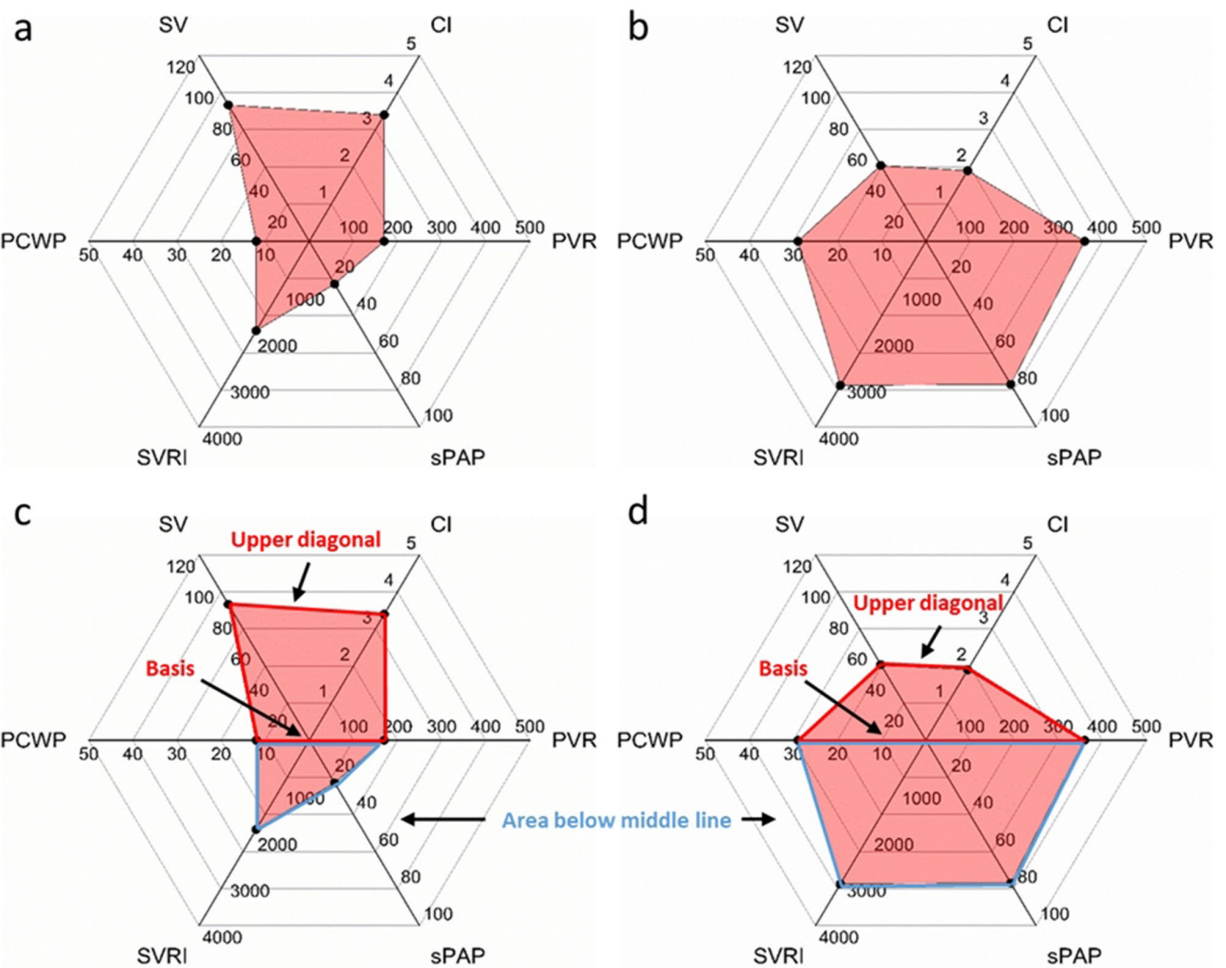
Hemodynamic profiling. Hemodynamic profiles. Panel a: healthy adult. Panel b: patient in cardiogenic shock. Hemodynamic profiles give an instant understanding of the hemodynamic situation and suggest treatment options. Panel c: same patient as in panel a. An upper diagonal longer that the length of the basis can indicate a compensated hemodynamic state. The blue borders define the area below the middle line. A small lower area in a compensated hemodynamic situation reflects normal status or optimal treatment. A small lower area in a state of decompensation can either indicate optimal treatment or the need for more vasopressor support, depending on the underlying cardiac pathology. Panel d: the base is longer than the upper diagonal, indicating cardiac stress or cardiac decompensation. The large filled area below the middle line indicates treatment options such as lowering PVR and/or sPAP. Effective treatment should increase the cardiac index and lengthen the upper diagonal in relation to the basis and reduce the filled area below the middle line. CI: cardiac index [L·min^-1^·m^-2^]; SV: stroke volume [mL]; PCWP: pulmonary capillary wedge pressure [mmHg]; SVRI: systemic vascular resistance index [dyn·s·cm^−5^·m^−2^]; sPAP: systolic pulmonary artery pressure [mmHg]; PVR: pulmonary vascular resistance [dyn·s·cm^−5^].

**Figure 2:**
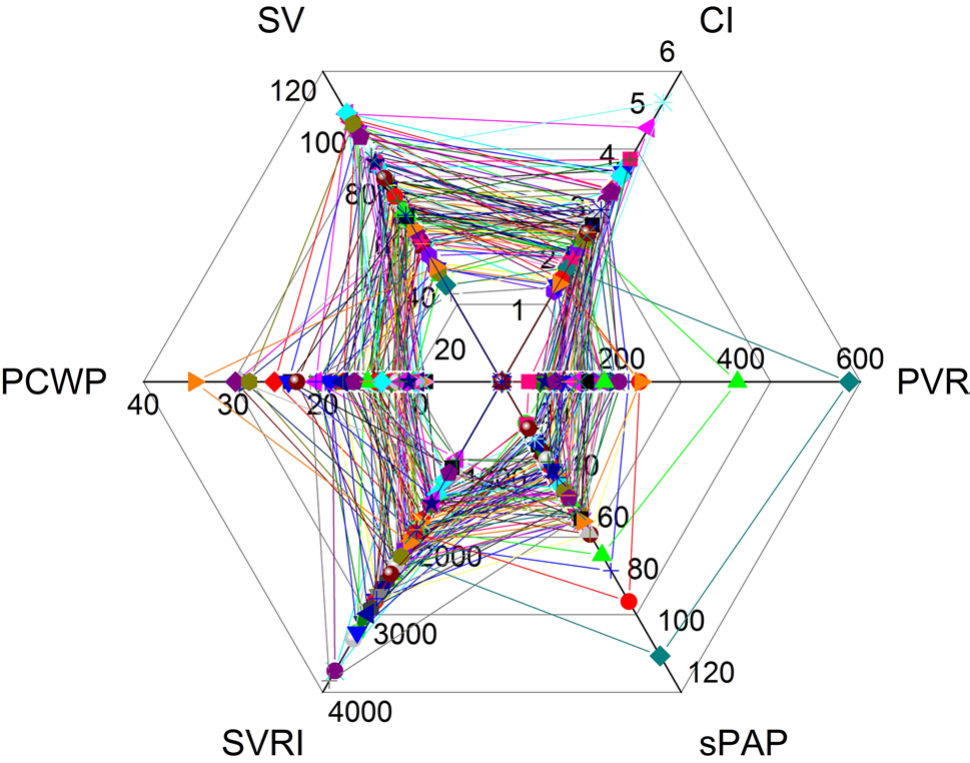
Combined hemodynamic profiles. Hemodynamic profiles of all 107 ICU patients on day three of their intensive care stay as determined by non-invasive hemodynamic critical care echocardiography. Although mean arterial pressure (MAP) was above 60 mmHg in 96.1% of patients, hemodynamic profiling revealed hemodynamic states that induced severe cardiac stress or implied acute cardiac decompensation. CI: cardiac index [L·min^−1^·m^−2^]; SV: stroke volume [mL]; PCWP: pulmonary capillary wedge pressure [mmHg]; SVRI: systemic vascular resistance index [dyn·s·cm^−5^·m^−2^]; sPAP: systolic pulmonary artery pressure [mmHg]; PVR: pulmonary vascular resistance [dyn·s·cm^−5^].

Determination of left ventricular ejection fraction (LVEF) was successful in 70.1% of patients using the Simpson method and in 97.2% using visual assessment techniques. Determination of right ventricular function was successful in 88.8% using tricuspid annular plane systolic excursion (TAPSE) and in 79.4% using tricuspid annular peak systolic velocity (S’). We were able to obtain valid CO and CI data in 96.3%, PCWP in 93.5%, PVR in 88.4%, sPAP in 89.7%, SVR and SVRI in 96.3%, LAP in 93.5%, and ratio of mitral inflow and the average early diastolic velocity of the mitral annulus obtained by tissue Doppler (E/e’) in 93.5% of patients. Quantitative hemodynamic data are plotted as web graphs (Fig. 1). The superimposed plots of all examined patients are shown in Fig. 2.

### Correlation between clinical parameters and echocardiographic non-invasive hemodynamics

In order to identify a surrogate parameter for hemodynamic compromise, the parameters determined by CCE and non-invasive hemodynamics (TAPSE, CO and CI), MAP, systolic blood pressure (sBP) and LVEF were analyzed. No clinically useful correlations were found (Table 2).

**Table 2:** Correlations between systolic and mean arterial pressures and hemodynamic parameters determined by echocardiography.

	*r*	*r*^2^	adj. *R*^2^	Equation
Correlation of MAP with CI	0.005	0.00003	0	CI = 2.979—(0.000267·MAP)
Correlation of MAP with LVEF	0.054	0.003	0	LVEF = 53.492 + (0.0498·MAP)
Correlation of MAP with TAPSE	0.032	0.001	0	TAPSE = 20.105 + (0.0152·MAP)
Correlation of sBP with CI	0.078	0.006	0	CI = 2.659 + (0.00235·sBP)
Correlation of sBP with LVEF	0.148	0.022	0.012	LVEF = 47.141 + (0.0823·sBP)
Correlation of sBP with TAPSE	0.231	0.053	0.043	TAPSE = 12.777 + (0.0674·sBP)
Correlation of LVEF with CI	0.268	0.072	0.063	CI = 2.113 + (0.0146·LVEF)
Correlation of LVEF with CO	0.289	0.084	0.075	CO = 3.832 + (0.0328·LVEF)

*MAP* mean arterial pressure, *CI* cardiac index [L·min^−1^·m^−2^], *LVEF* left ventricular ejection fraction [%], *TAPSE* tricuspid annular plane systolic excursion [mm], *sBP* systolic blood pressure [mmHg], *CO* cardiac output [L·min^−1^], *r* correlation coefficient, r^2^ coefficient of determination, *adj. R*^*2*^ adjusted R-squared value.

### Tricuspid regurgitation in the presence of PiCCO or PA catheters

Significant tricuspid regurgitation (TR) was present in 15.9% of the patients, graded as moderate in 10.3% and severe in 5.6%. Invasive hemodynamic monitoring was used in 24.3% of the patients (PiCCO in 22.4%; PAC in 1.9%). Significant TR was present in 23.1% of the patients who had invasive hemodynamic monitoring. In these patients with significant TR, the close correlation between thermodilution and echocardiography observed in patients without TR could no longer be detected (Supplementary Table S1).

### Tricuspid annular plane systolic excursion and tricuspid annular peak systolic velocity measurements

**Figure 3:**
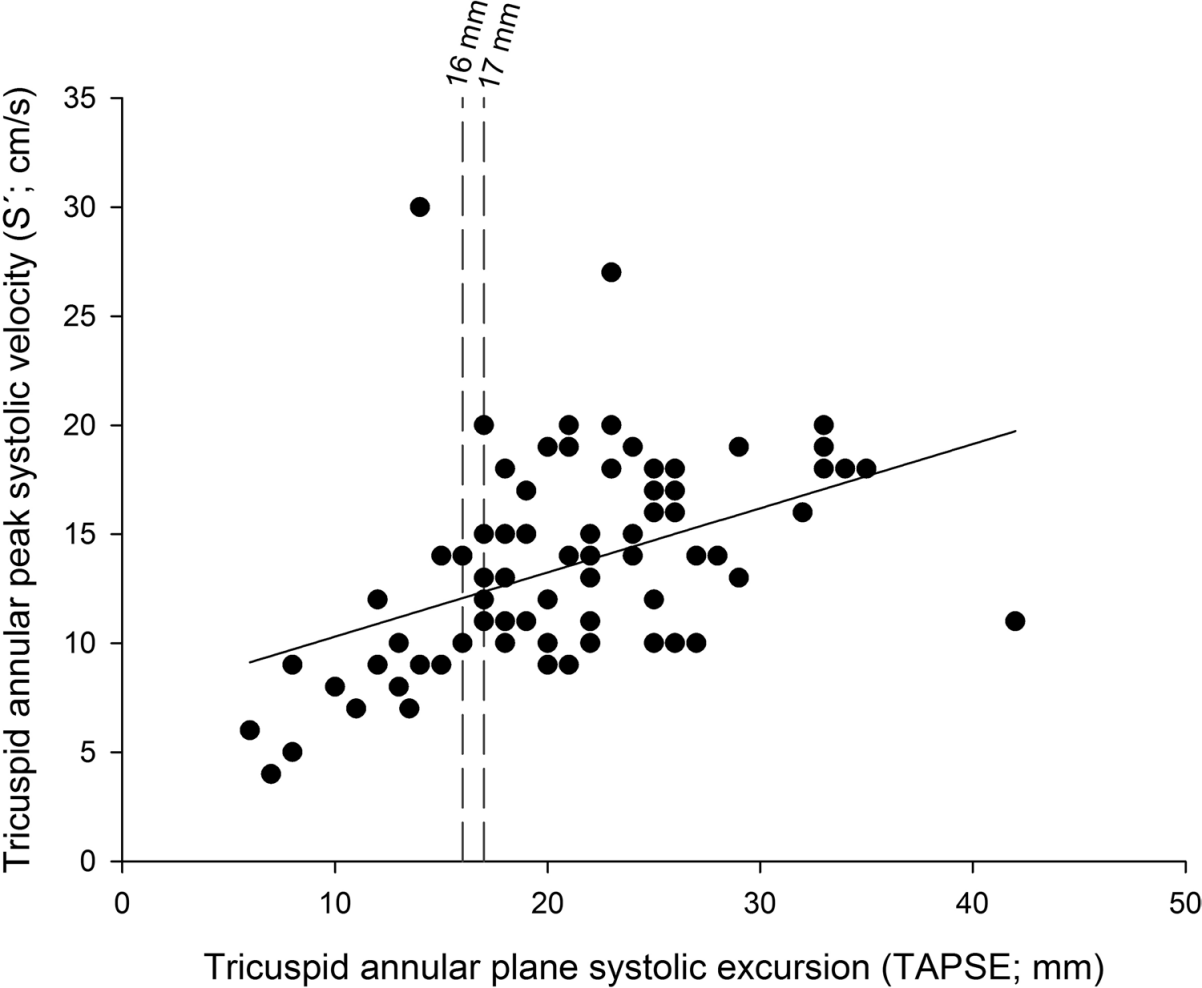
Linear regression analysis of tricuspid annular plane systolic excursion (TAPSE) and tricuspid annular peak systolic velocity (S’).

We were able to measure both parameters in 84 of the 107 patients (78.5%). The correlation coefficient (*r*) between TAPSE and S’ was 0.43 in the critical care setting (Fig. 3, Supplementary Table S2). When evaluating cut-off values for right ventricular dysfunction (RVdys), S’ predicted RVdys in 73.3% of the cases according to the 2010 ASE guidelines and in 64.7% according to the 2015 ASE guidelines when TAPSE was taken as the gold standard. In our study population, the probability of TAPSE predicting normal right ventricular function when the S’ value indicated RVdys was 21.4% (2010 and 2015 ASE guidelines; Supplementary Table S2).

Linear regression analysis of TAPSE and S’. Regression equation: S' = 7.357 + (0.295·TAPSE). Vertical dashed lines show the TAPSE threshold values for right ventricular dysfunction (16 mm for 2010 American Society of Echocardiography guidelines and 17 mm for 2015 American Society of Echocardiography guidelines).

### Value of routine hemodynamic CCE analysis

**Figure 4:**
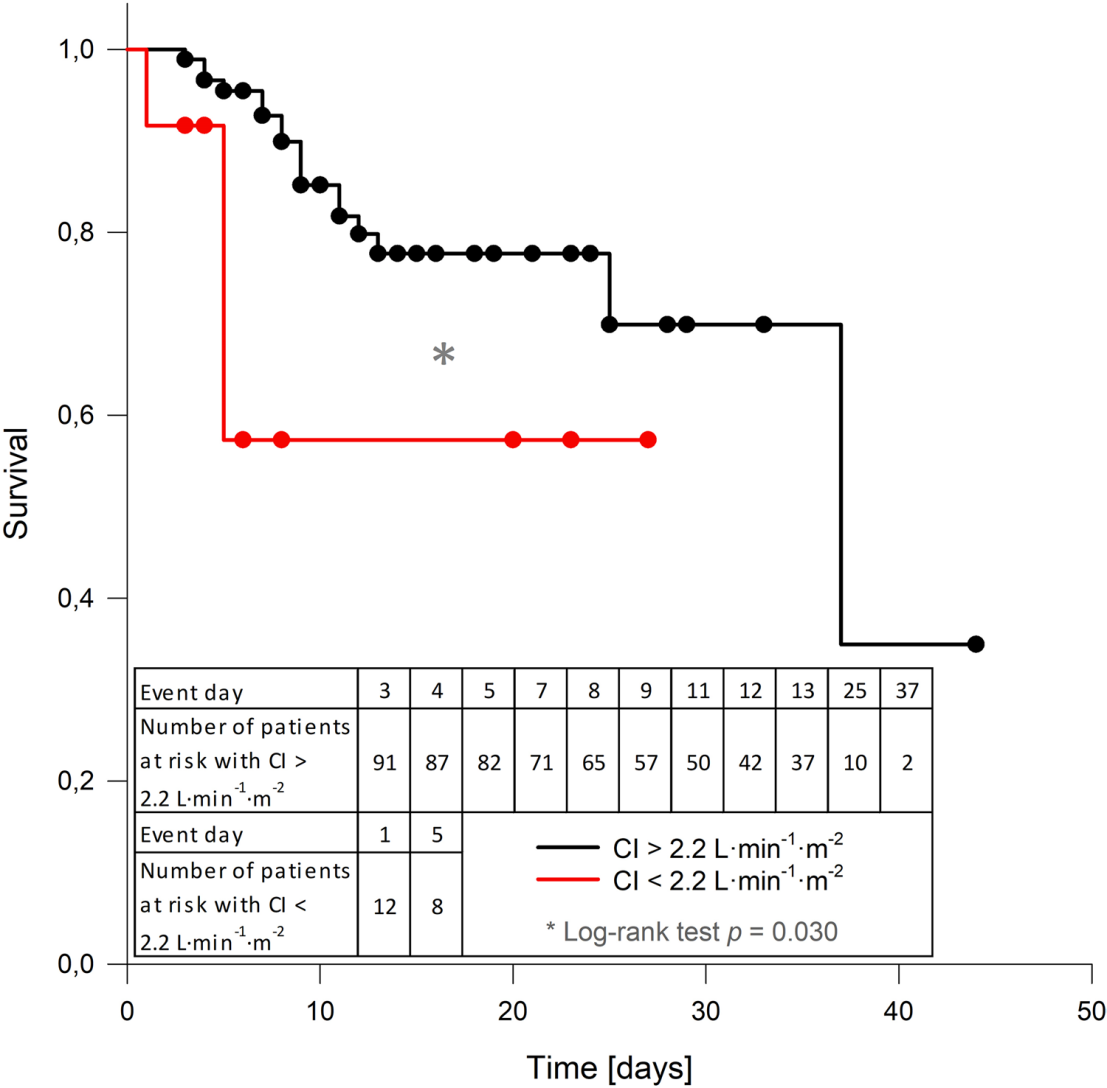
Kaplan–Meier survival curves.

Our study was not designed with survival as a primary endpoint. However, our study patients with a CI greater than 2.2 L·min^−1^·m^−2^ had a predicted mortality of 14.6% (individual mortality prediction according to individual SAPS II scores) and an actual ICU mortality of 18.7%. Patients with a CI less than 2.2 L·min^−1^·m^−2^ had a predicted mortality of 12.7% (according to individual SAPS II scores) while the actual ICU mortality was 27.3%. Log-rank analysis of the Kaplan–Meier curves also showed a statistically significant ICU survival disadvantage for patients with a CI below 2.2 L·min^−1^·m^−2^ (Fig. 4).

Kaplan–Meier curves of patients with a cardiac index (CI) above and below 2.2 L·min^−1^·m^−2^. A statistically significant difference in ICU survival times between both groups was determined by log-rank analysis (*p* = 0.030). Dots show right-censoring.

## Discussion

Although echocardiography-based hemodynamic assessments are mentioned in guidelines and recommendations^26–30^, CCE has not yet found its way into routine clinical practice^7^, and hemodynamic parameters are mostly determined by qualitative visual evaluation rather than by objective, quantitative methods.

Despite the known limitations of CCE image quality in ICU patients, it can be employed in the vast majority of ICU patients when performed by an expert examiner^14^. Limitations apply to patients with absent or very restricted transthoracic imaging windows.

The descriptive and comparative results of our prospective observational study have closed the gap between the classical descriptive echocardiographic and the hemodynamic assessment of the individual patient by extending CCE to include quantitative hemodynamic parameters that were previously the domain of invasive hemodynamic monitoring techniques.

This offers the opportunity to directly evaluate the cause of cardiovascular deterioration, for example a drop in sBP or MAP. Therefore, if a decrease in CI were detected by non-invasive hemodynamic CCE, it could now be further attributed for example to high PVR and/or diastolic dysfunction without the need for invasive hemodynamic measurements. The detected underlying pathology could thus then be subjected to specific treatment instead of simply reporting deteriorating hemodynamic values. Furthermore, quantitative hemodynamic parameters such as PCWP, CI and SVR can be used in real time and individually to evaluate the true hemodynamic impact of standard descriptive echocardiographic parameters such as a decreased LVEF or valvular disease.

Visualizing the hemodynamic results in six-dimensional web graph plots (hemodynamic profiling) might help intensivists to identify targets for hemodynamic improvement and specific treatment (Fig. 1). Considering all filled areas below the middle line of the plot as hemodynamically destabilizing and potentially harmful, targets for hemodynamic improvement can now be identified even by less experienced intensivists. Visualizing data for better interpretation results is an accepted and self-evident principle^31–33^. The effects of altering treatment can likewise easily be extracted from the area above the middle line of the plot, the length of whose base should not exceed that of the upper diagonal (Fig. 1c,d).

Direct hemodynamic assessments are essential in critically ill patients, because MAP, sBP and other clinical parameters used as surrogate markers cannot be used to determine CI in most critical care scenarios^34^ (Table 2). Although MAP was above the threshold value of 60 mmHg in 96.1% of all patients, CI was less than 2.5 L·min^−1^·m^−2^ in 26.2% and less than 2.2 L·min^−1^·m^−2^ in 10.7%. These findings highlight the gap between clinically perceived and actual individual hemodynamics in ICU patients.

Predicted (based on individual SAPS II score mortality estimation) and actual ICU mortality rates in patients with a CI above 2.2 L·min^−1^·m^−2^ did not significantly differ. In our institution, even outside the current study cohort, SAPS II predicted mortality rates and actual ICU mortality rates are very close^35^. However, in our study patients with a low CI, actual ICU mortality rates were more than two-fold higher than the mortality rate predicted by the SAPS II scores. This identifies CI as a potential risk factor for mortality in the ICU. Kaplan–Meier and log-rank analysis likewise revealed statistically significant lower survival rates in patients with a low CI while in the ICU (Fig. 4). We interpret the lower probability of survival for patients with a lower CI as a further indication for the need for routine hemodynamic assessments with implementation of appropriate therapy adjustments, although hard evidence that this improves clinical outcome is still lacking^14,36^.

The current study was not designed as a comparison study between invasive and non-invasive hemodynamic evaluation methods. However, the need for routine non-invasive hemodynamic CCE or at least CCE alone was even more evident in our patient cohort, because 23.1% of PAC or PiCCO measurements were obtained in patients with significant TR, potentially resulting in invalid data^8,9^. Without CCE, these data would have inevitably led to misinterpretation of the clinical situation; similar patients may have negatively influenced the results of previous studies evaluating thermodilution techniques. There are also other conditions that can negatively affect the accuracy of hemodynamic measurements with PACs and thermodilution techniques (e.g., arrhythmias, pulmonary edema)^8–10^, and these might further increase the risk of invalid measurements to greater than the 23.1% estimated above.

Systematic echocardiographic examinations and non-invasive hemodynamic CCE assessments additionally revealed that the confirmed high correlation TAPSE and S’ in non-ICU patients as parameters of longitudinal right ventricular function could not be reproduced in ICU patients (Fig. 3, Supplementary Table S2). There is already some evidence that the correlations of parameters found in diastolic dysfunction in non-ICU patients are not valid in ICU patients^18^. The low correlation between the longitudinal right ventricular function parameters TAPSE and S’ in this study highlights the fact that many echocardiographic parameters and classifications have not been evaluated in ICU patients; correlating the echocardiographic findings with hemodynamic parameters in critically ill patients would help to assess their true impact on cardiac function in this subgroup.

### Limitations of the study and caveats

Several limitations and caveats apply to our prospective observational study, which yielded a moderate amount of descriptive data. Compared to invasive hemodynamic monitoring, assessment using hemodynamic CCE requires extensive knowledge and training in echocardiography, which is currently difficult to provide to a sufficient number of non-cardiologist intensivists, and having skilled practitioners available on a 24/7 basis is even more difficult. Non-invasive hemodynamic CCE is non-continuous compared to a number of invasive methods that provide continuous data. In previous studies, non-invasive hemodynamic parameters correlated sufficiently well with invasive measurements to allow the former to be used clinically, and for absolute values to be reported. For example, the correlation coefficient between invasive and non-invasive measurements of CO is 0.94 with a standard deviation of 8.6%^14^, and for PCWP the correlation coefficient is 0.87^20^ with a difference between Doppler and catheter measurements of 0.1 ± 3.8 mmHg^20^. However, SV estimation with echocardiography can be affected and limited by several geometric assumptions regarding the LVOT assessment. There is conflicting evidence that some hemodynamic parameters are affected by underlying cardiac abnormalities and under certain conditions, measurements may be of limited value. For example, the PCWP estimated by the Nagueh formula^22^ is probably not well correlated with the invasively measured PCWP in patients with normal LVEFs, left bundle branch block or mitral regurgitation^37^. Therefore, single measurements should always be interpreted with care, and multiple readings over time are preferred, especially in ICU patients, until further evidence becomes available.

Survival was not a primary endpoint parameter in this study. Therefore, the observed survival benefit must be interpreted with care. Without a control group, the study results are only descriptive. In addition, specific limitations for the use of echocardiographic assessments apply to critically ill patients and those with multivalvular disease. For example, the presence of mitral regurgitation influences tricuspid regurgitation with a likely increase in PAP and a consequent impact on the regurgitant volume. Severe regurgitant lesions, such as severe TR, may also impact on sPAP approximations or functional measurements such as TAPSE and S’^38^. However, the impact of these inaccuracies is not always quantifiable. Therefore, multiple evaluations with different approaches should be performed and single readings interpreted with care.

Hemodynamic measurements are not solely the domain of thermodilution techniques and echocardiography, and can also be acquired by other techniques (e.g., bioimpedance, waveform analysis). These techniques are not discussed here but can be valuable additions or alternatives in specific situations^39^.

## Conclusions

The mainly descriptive and partly comparative results of the current study indicate that hemodynamic CCE could be used in the vast majority of ICU patients to determine the hemodynamic impact of the structural changes detected by standard echocardiography and to identify dedicated targets for improving deteriorating hemodynamic states. Hemodynamic profiling could aid this process by visualizing the current hemodynamic state and simplifying target identification for specific target-directed treatments. Hemodynamic CCE could be especially advantageous when conditions are present in which thermodilution methods might produce invalid results. By the use of CCE, a subset of patients with formerly unknown low cardiac indices that have a higher mortality while in the ICU could be identified.

## Supplementary Information

Supplementary Information 1.

Supplementary Information 2.

## Data Availability

The datasets generated during and/or analyzed during the current study are available from the corresponding author on reasonable request.
