# Supplementary material for: Hemodynamic profiling by critical care echocardiography could be more accurate than invasive techniques and help identify targets for treatment
Source: Sci Rep. 2022 May 3;12:7187. doi: 10.1038/s41598-022-11252-2 (PMC9065036; doi:10.1038/s41598-022-11252-2)
Supplement: Supplementary file 1 — Supplementary Information 1. [file 41598_2022_11252_MOESM1_ESM.pdf]

Supplementary Table S1.

Number of patients with tricuspid regurgitation, and with PAC or PiCCO® catheter

Moderate tricuspid valve insufficiency	11 (10.3)
Severe tricuspid valve insufficiency	6 (5.6)
Modere or severe tricuspid valve insufficiency	17 (15.9)
PiCCO®	24 (22.4)
PAC	2 (1.9)
PiCCO® or PAC	26 (24.3)
**Patients with PiCCO® or PAC and, moderate or severe tricuspid valve insufficiency**	**6 (23.1)**

Number of patients and (percentage). PAC: pulmonary artery catheter; PiCCO®: pulse contour cardiac output system (Pulsion Medical Systems)
