# Supplementary material for: Hemodynamic profiling by critical care echocardiography could be more accurate than invasive techniques and help identify targets for treatment
Source: Sci Rep. 2022 May 3;12:7187. doi: 10.1038/s41598-022-11252-2 (PMC9065036; doi:10.1038/s41598-022-11252-2)
Supplement: Supplementary file 2 — Supplementary Information 2. [file 41598_2022_11252_MOESM2_ESM.pdf]

Supplementary Table S2.

Correlation of tricuspid annular plane systolic excursion with tricuspid annular peak systolic velocity

Correlation of TAPSE with S'<br>Equation	R = 0.432 R^2 = 0.187 adj. R^2 = 0.177<br>S' = 7.357 + (0.295 · TAPSE)
Probability of S' <10 $cm \cdot s^{-1}$ if TAPSE <16 mm	73.3%
Pobability of S' <10 $cm \cdot s^{-1}$ if TAPSE <17 mm	64.7%
Probability of TAPSE >16 mm if S' <10 $cm \cdot s^{-1}$ or <9.5 $cm \cdot s^{-1}$	21.4%
Probability of TAPSE >17 mm if S' <10 $cm \cdot s^{-1}$ or <9.5 $cm \cdot s^{-1}$	21.4%

Correlation of tricuspid annular plane systolic excursion (TAPSE) with tricuspid annular peak systolic velocity (S') in intensive care patients (n = 84). R: correlation coefficient; R^2: coefficient of determination; adj. R^2: adjusted R-squared value
